# Intercalary prosthetic replacement is a reliable solution for metastatic humeral shaft fractures: retrospective, observational study of a single center series

**DOI:** 10.1186/s12957-021-02250-1

**Published:** 2021-05-05

**Authors:** Zhiqing Zhao, Zhipeng Ye, Taiqiang Yan, Xiaodong Tang, Wei Guo, Rongli Yang

**Affiliations:** Musculoskeletal Tumor Center, Peking University People’s Hospital, Beijing, 100044 China

**Keywords:** Metastasis, Humeral shaft, Intramedullary nailing, Plate fixation, Intercalary prosthesis

## Abstract

**Background:**

Treatments for metastatic fracture of the humeral shaft continue to evolve as advances are made in both oncological and operative management. The purposes of this study were to critically evaluate the effectiveness of intercalary endoprostheses in treating metastatic humeral shaft fractures and to clarify the surgical indications for this technique.

**Methods:**

Sixty-three patients treated surgically for 66 metastatic fractures of the humerus shaft were retrospectively reviewed. Intramedullary nailing (IMN) was performed in 16 lesions, plate fixation (PF) in 33 lesions, and prosthetic replacement in 17 lesions. The operative time, intraoperative blood loss, and postoperative complications were noted. The function of the upper extremities was assessed by the Musculoskeletal Tumor Society (MSTS) score and American Shoulder and Elbow Surgeons (ASES) score. All included patients were followed until reconstructive failure or death.

**Results:**

The operative time was relatively shorter in the prosthesis group than in either the IMN group (*p* = 0.169) or PF group (*p* = 0.002). Notably, intraoperative blood loss was significantly less in the prosthesis group than in either the IMN group (*p* = 0.03) or PF group (*p* = 0.012). The average follow-up time was 20.3 (range, 3–75) months, and the overall survival rate was 59.7% at 12 months and 46.7% at 24 months. One rotator cuff injury, 3 cases of iatrogenic radial nerve palsy, 5 cases of local tumor progression, and 1 mechanical failure occurred in the osteosynthesis group, whereas one case of aseptic loosening of the distal stem and one case of local relapse were observed in the prosthesis group. There were no significant differences in functional scores among the three groups.

**Conclusions:**

Intercalary prosthetic replacement of the humeral shaft may be a reliable solution for pathologic fractures patients; it is indicated for lesions with substantial bone loss, or accompanied soft tissue mass, or for those patients with better prognosis.

**Supplementary Information:**

The online version contains supplementary material available at 10.1186/s12957-021-02250-1.

## Introduction

The humerus is the second most common long bone affected by metastatic diseases, following the femur, and the incidence of pathologic fractures has been reported to range from 16 to 27% [1–3]. Conservative treatment does not lead to significant bone healing because the tumor cells grow rapidly and overwhelm the bone’s reparative process [4]. Radiotherapy alone can partially relieve pain but may further delay bone healing and the restoration of function [5, 6]. Consequently, a growing number of studies have suggested that patients with metastatic humeral shaft fractures benefit from surgical stabilization [7–10].

Different surgical options have been described in previous studies [7, 11–15]. Intramedullary nailing (IMN) can be carried out by a closed or open procedure [12], but it may cause rotator cuff injury, whereas plate fixation (PF) requires extensive stripping of the soft tissues from the bone [16]. During the last decade, tyrosine kinase inhibitors, in particular gefitinib, sorafenib, and bortezomib, have been introduced into routine practice for first-line treatment against advanced nonsmall cell lung cancer, renal cell carcinoma, and multiple myeloma, and the survival of these metastatic patients has been considerably improved owing to these latest oncological treatments. Many cancer surgeons prefer more aggressive treatments to provide lasting palliation. This has led to the application of surgical techniques used for the treatment of primary sarcomas in bone [17, 18]. Therefore, when possible, more radical treatment is needed for some patients with solitary bone lesions and a longer predicted life expectancy. Encouraging results regarding the application of intercalary prostheses have been obtained from studies on intercalary prostheses, leading to the expansion of surgical indications and controversy regarding the treatment of choice [13, 19, 20]. Additionally, mechanical studies conducted in vitro have indicated that prostheses provide a stronger construct than IMN does in a model of humeral segmental defects [21, 22].

This study is a retrospective evaluation of a single center experience of 66 humerus shaft fractures treated by IMN, PF, and intercalary prosthetic reconstruction. The aims were to offer adequate individual treatment to the patient and try to clarify the indications for prosthetic surgical procedures. Our hypothesis was that prosthetic replacement can reduce the operative time, intraoperative blood loss, and postoperative complications more than intramedullary nailing or plate fixation.

## Materials and methods

### Inclusion and exclusion criteria

This retrospective study was approved by the Ethical Review Committee (ERC), and the requirement for informed consent from all subjects was waived. We retrospectively reviewed the patients between 2005 and 2018. The inclusion criteria were as follows: (i) patients with pathological fractures of the humeral diaphysis due to a metastatic disease, (ii) patients who received surgical treatment, and (iii) patients with available demographic and medical records. The exclusion criteria were (i) a fracture caused by primary malignant or benign bone tumors, (ii) involvement of the articular surface or entire humerus, and (iii) a history of revision of the initial surgery performed at an outside institution.

### Patient characteristics

**Fig. 1:**
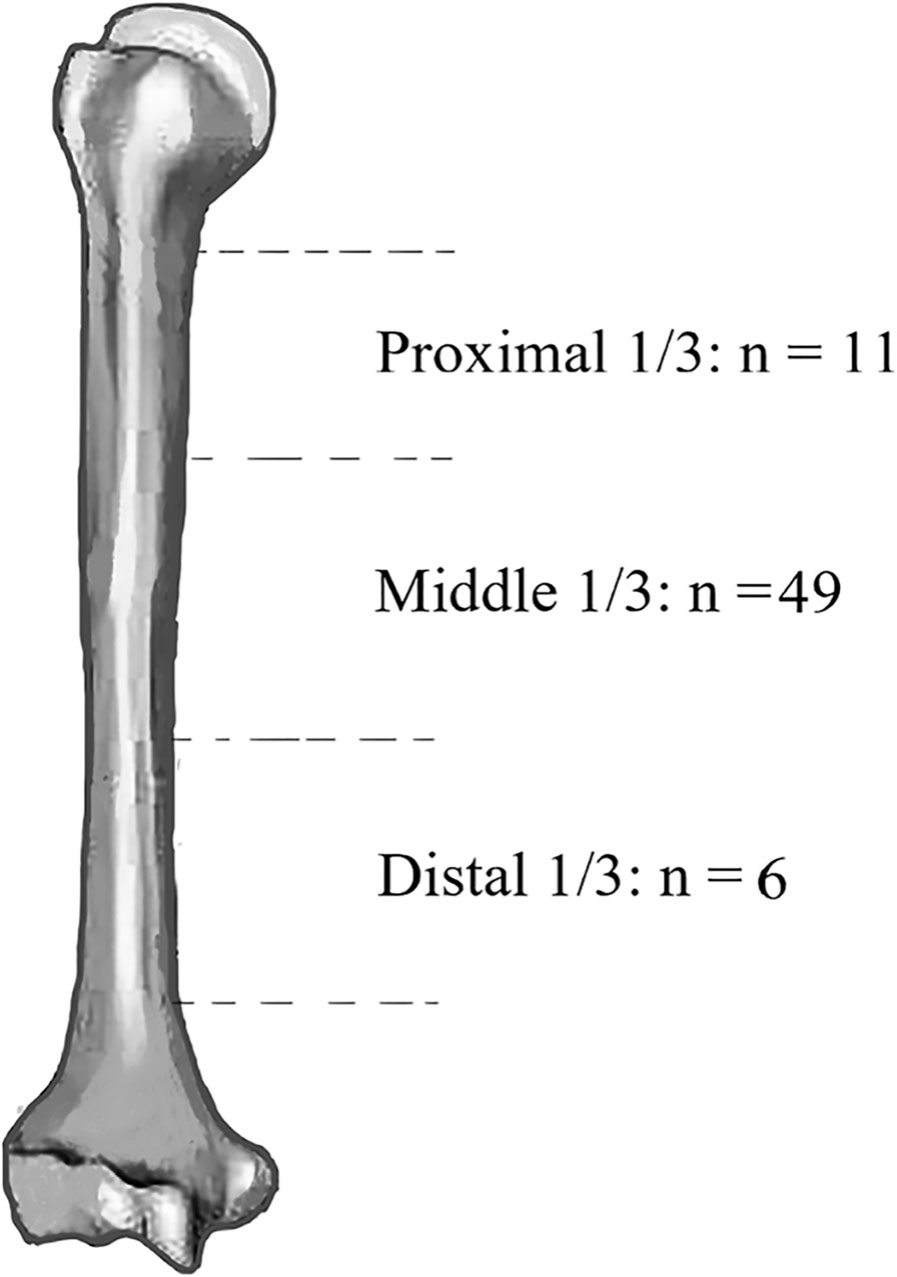
The distribution of lesions in the humeral shaft

According to the aforementioned criteria, sixty-three patients were included. Bilateral humeral shaft fractures were observed in 3 patients, leading to a total of 66 procedures. All surgical procedures were performed by the same team of trained orthopedic oncology surgeons. Among the methods used for definitive treatment, the most prevalent was open reduction and internal fixation with plates and screws, followed by intercalary prostheses and IMN, and these methods were performed for 33 (50%) cases, 17 cases, and 16 cases, respectively. Before 2012, only IMN and PF were used, but since 2012, intercalary prostheses have been used in select patients in an attempt to improve disease control; however, osteosynthetic techniques have continued to be used. The study population comprised 33 males and 30 females, with a mean age of 62.3 ± 10.7 years (range, 39–82 years) at diagnosis (Table 1). The mean interval between the diagnosis of the primary tumor and the development of humeral shaft metastasis was 13.5 ± 25.6 months (range, 0–120 months). The primary lesions were myeloma in 18 cases, lung cancer in 18, renal cancer in 11, breast cancer in 4, thyroid carcinoma in 2, and other types in 10. Metastatic lesions were located at the upper, middle, and lower 1/3 of the humerus diaphysis in 11, 49, and 6 cases, respectively (Fig. 1). A total of 41 patients had multiple bone metastases. Complete pathologic fractures were seen in 40 lesions, and accompanying soft-tissue masses were observed in 29 lesions. The average Mirel’s score in patients with impending fractures was 8.4, and the scores ranged from 8 to 9. The presence of visceral metastases was found in seventeen patients (27.0%, 17/63). All patients suffered from severe pain and dysfunction of the arm, which severely affected their ability to perform activities of daily living. Twenty patients (31.7%, 20/63) received chemotherapy and/or radiotherapy for primary lesions before surgery. Only one patient received radiotherapy for humeral metastatic lesions. More details about the type of fracture, presentation of visceral metastases, Karnofsky performance score (KPS), visual analog scale (VAS) score, Mirel’s score, and TNM stage are exhibited in Supplementary Table I.

**Table 1:** Patients and treatment characteristics

Variable	No. [%]
Operations	66
Patients	63
Male	33 [52]
Female	30 [48]
Mean age (years)	62.3 (SD, 10.7; range, 39-82)
Primary tumor	
Lung cancer	18 [29]
Myeloma	18 [29]
Renal carcinoma	11 [17]
Breast cancer	4 [6]
Thyroid	2 [3]
Others	10 [16]
Karnofsky score	68.9 (range, 30–90)
Mirel’s score	8.4 (range, 8–9)
Skeletal metastasis	
Solitary	22 [35]
Multiple	41 [65]
Type of fracture	
Complete	40 [61]
Impending	26 [39]
Accompanied soft tissue mass	
Yes	29 [44]
No	37 [56]
Visceral metastases	
Yes	17 [27]
No	46 [73]
Surgery	
IMN	16 [24]
PF	33 [50]
Prosthesis	17 [26]

Perioperative events were noted regarding the operating duration (min), intraoperative blood loss (ml) and postoperative complications. At the final follow-up, a postoperative functional assessment was carried out using the American Shoulder and Elbow Surgeons (ASES) [23] and Musculoskeletal Tumor Society (MSTS) scales [24]. All included patients were followed until reconstructive failure or death.

### Surgical options

Closed reduction and stabilization with anterograde unreamed locked nail insertion were performed in patients with bilateral humeral shaft fractures, and poor survival was estimated. Open IMN or plates are acceptable when adequate proximal and distal cortical bone is present for screw purchase. Additionally, preoperative radiographs can be used to determine whether a very narrow intramedullary canal requiring plating is present. Our general indications for intercalary prosthetic replacement were solitary hypervascular metastatic lesions, lesions with large cortex destruction, and distinct soft tissue masses. The prerequisite for performing this procedure is that approximately 5 cm of the intramedullary canal remains after segmental resection at each end to accept the implant stem.

### Surgical technique

#### Intralesional procedure and reinforced osteosynthesis

Most commonly, a brachialis-splitting approach was used to expose the anterior aspect of the humerus [25]. A cortical window was created through the area of bone destruction, and all of the gross tumors were completely curetted.

If the humerus was prepared to receive IMN, the entry point was made via the delta-split approach following the standard antegrade nail insertion technique [26]. An appropriately sized nail was inserted over a guide wire, and the length and fracture reduction of the entire humerus were verified. Then, the nail was locked proximally and distally to provide immediate rotational stability and avoid telescoping. Finally, polymethylmethacrylate containing gentamicin was added to the tumor cavity.

Before the plate was applied, reduction of the fracture and implantation of the internal fixation device were performed. The screws were removed temporarily, except for the screws at the proximal and distal ends of the plate. Additionally, bone cement was introduced into the proximal and distal fragments via a cement gun, and then, the removed screws were reinstalled before the cement hardened. Therefore, the intramedullary cement and the screws became a solid “reinforced concrete structure.”

#### Wide resection and segmental endoprosthesis reconstruction

**Fig. 2:**
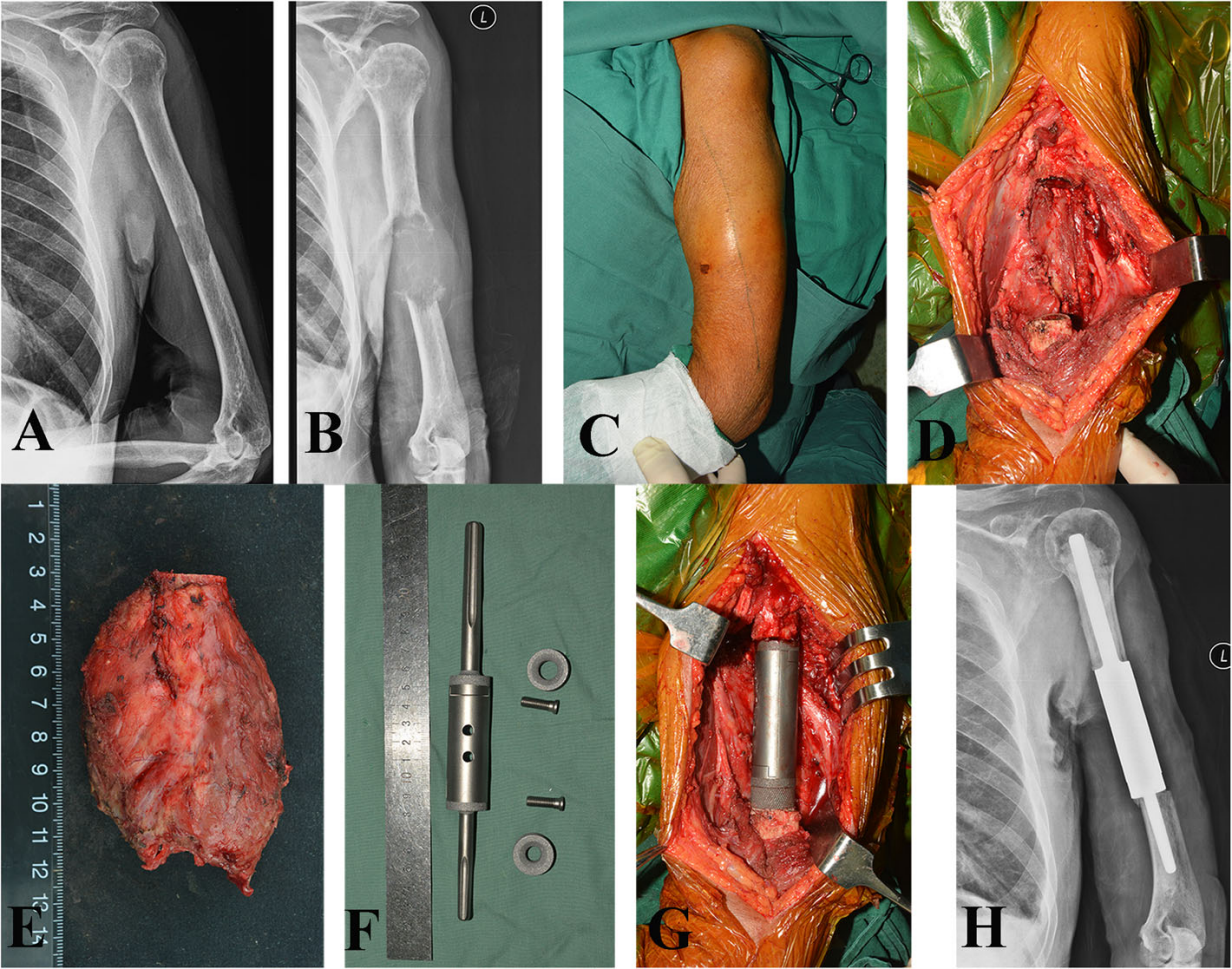
Intercalary prosthetic reconstruction for a pathologic fracture of the left humeral shaft in patient 59, a 76-year-old male. **a** Anteroposterior radiograph of the left humerus showed osteolytic destruction in the middle 1/3 of the humerus. **b** The metastatic lesion of lung carcinoma was enlarged despite 3 months of conservative treatment. **c** The incision was made proximally through a deltopectoral approach and distally on the arm over the medial border of the biceps muscle. **d**, **e** Intraoperative photos show that the lesion was resected widely. **f**, **g** Intraoperative photos show the custom-made prosthesis. **h** Postoperative X-ray images show that the prosthesis is in a good position

Wide intercalary resection of the humeral diaphyseal lesions was performed according to the Ennecking principle [27]. The proximal and distal canals were reamed to accommodate the stems. The prosthesis was manufactured using computer-aided design and manufacturing technologies after the level of humeral transection was determined (CHUNLi Corp., Beijing, China). Intraoperative fluoroscopy was used to monitor the reaming process to avoid penetration into the adjacent joint. Facing reamers were used to machine the proper radius of curvature of the exposed cortical bone to ensure a flush fit of the implant’s stem-body junction. The intramedullary stems were simultaneously fixed at the proximal and distal bone stumps with cement via a cement gun. Then, the spacer was assembled in situ and connected using two locking bolts (Fig. 2).

### Postoperative treatment

Palliative chemotherapy or targeted therapy was administered based on the specific tumor type and treatment protocol practiced at that time. External beam radiation was used or was planned for almost all intralesional surgical interventions. Generally, radiotherapy was implemented approximately 4 weeks postoperatively. Intravenous bisphosphonates were routinely used for all cases.

### Statistical analysis

The data are presented as the mean ± standard deviation (SD). Statistical analysis was performed using SPSS software version 22 (IBM Corp., Armonk, New York, USA). One-way ANOVA was utilized to assess the differences among the three groups in age, operating duration, blood loss, implant follow-up period, and functional scores. A post hoc test was then performed to evaluate the ANOVA results. The rates of complications among the three groups were compared by the chi-square test (or Fisher’s exact test).

## Results

**Fig. 3:**
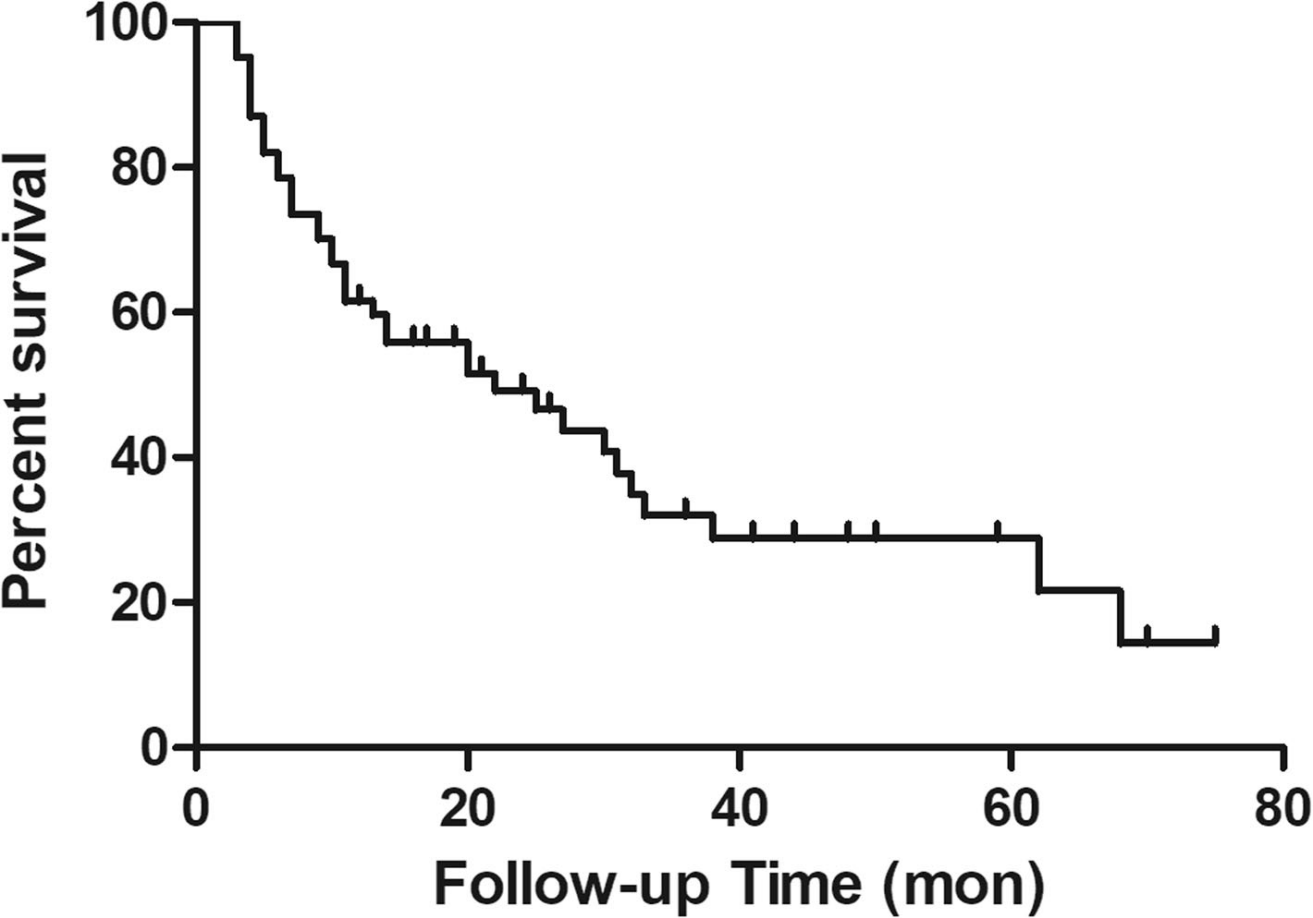
The Kaplan-Meier curve of overall survival probability for all patients

The average follow-up time was 20.3 ± 18.4 months (range, 3–75 months). The overall survival rate was 59.7% at 12 months and 46.7% at 24 months (Fig. 3). At the latest follow-up, 38 patients had died of tumor progression or visceral metastasis (Table 2).

**Table 2:** Details of patient and tumor characteristics, complications, and functional outcomes

No.	Age/Gender	Primary tumor	Operation	Operative time (min)	Estimated blood loss (ml)	Follow-up time (month)	Complications	MSTS score	ASES score
1	68/F	Lung cancer	IMN	120	500	4	Shoulder joint impingement	19	63
2	78/M	Lymphoma	IMN	170	600	36		25	83
3	80/F	Myeloma	IMN	130	500	5		21	70
4	72/M	Renal cancer	IMN	180	2500	62	LR (11 months)	24	80
5a	65/F	Myeloma	IMN	100	200	30		24	80
5b			IMN	110	300			21	70
6	75/F	Lung cancer	PF	95	400	7		21	70
7a	62/M	Myeloma	IMN	90	200	4		20	67
7b			IMN	115	230		LR/FX (1 month)	21	70
8	70/M	Lung cancer	PF	95	300	6		23	77
9	55/M	Unknown	IMN	135	700	3		24	80
10	45/M	Gastric cancer	IMN	105	550	5		21	70
11	40/M	Lung cancer	IMN	125	650	7		23	77
12	53/M	Lung cancer	PF	145	300	9	Screw breakage (5 months)	20	67
13	44/F	Malignant peripheral schwannoma	PF	140	1600	13		22	73
14	71/M	Lung cancer	PF	155	400	4	RNI	20	67
15	47/F	Breast cancer	PF	150	200	13		23	77
16	79/M	• Prostate cancer	PF	135	300	14		22	73
17	52/M	Lung cancer	PF	205	200	5		26	87
18	47/F	Cervical cancer	PF	115	400	9		25	83
19	66/F	Myeloma	PF	80	800	10		20	67
20	58/F	Lung cancer	Prosthesis	105	150	6		24	80
21a	53/F	Malignant peripheral schwannoma	IMN	100	150	3		20	67
21b			IMN	90	100			21	70
22	76/F	Renal cancer	Prosthesis	150	300	70		20	67
23	60/M	Renal cancer	Prosthesis	95	400	75	LR (48 months)	20	67
24	75/M	Renal cancer	Prosthesis	100	100	68		23	77
25	61/M	Lung cancer	PF	245	800	10	LR/Fixation failure (6 months)	22	73
26	43/M	Unknown	Prosthesis	95	100	11		22	73
27	49/F	Myeloma	PF	120	200	59		25	83
28	60/M	Renal cancer	PF	150	200	22		23	77
29	71/F	Breast cancer	Prosthesis	195	150	33	Aseptic loosening (36 months)	20	67
30	70/F	Myeloma	PF	110	300	24		25	83
31	65/M	Lung cancer	Prosthesis	120	200	4		25	83
32	50/F	Myeloma	Prosthesis	100	100	20		24	80
33	45/F	Myeloma	Prosthesis	100	500	48		26	87
34	78/M	Pancreatic cancer	Prosthesis	65	200	14		20	67
35	60/M	Renal cancer	Prosthesis	120	300	31		23	77
36	78/F	Thyroid cancer	PF	165	800	41		24	80
37	61/F	Breast cancer	Prosthesis	135	100	50		24	80
38	62/F	Thyroid cancer	PF	160	500	38	RNI	19	63
39	66/M	Myeloma	PF	190	350	25		22	73
40	60/F	Lung cancer	PF	190	500	44		20	67
41	66/M	Myeloma	PF	125	650	20		23	77
42	39/F	Breast cancer	PF	135	800	32		24	80
43	61/F	Myeloma	Prosthesis	90	200	26		26	87
44	52/M	Renal cancer	PF	200	1000	27		23	77
45	66/F	Renal cancer	PF	180	600	17		25	83
46	73/M	Myeloma	PF	140	100	4		26	87
47	65/F	Lung cancer	PF	160	700	26		23	77
48	68/F	Myeloma	IMN	230	300	24		26	87
49	60/M	Renal cancer	PF	185	150	13		22	73
50	62/M	Myeloma	PF	180	1200	21		21	70
51	59/F	Myeloma	PF	140	1000	21		25	83
52	63/M	Renal cancer	Prosthesis	160	300	11		22	73
53	68/M	Myeloma	PF	190	1200	19		21	70
54	59/M	Lung cancer	PF	180	200	7	LR (4 months)	22	73
55	57/M	Lung cancer	PF	150	300	16		21	70
56	57/F	Rectal cancer	PF	180	200	11	RNI	22	73
57	52/F	Myeloma	IMN	120	550	12		21	70
58	67/F	Lung cancer	IMN	240	600	11		24	80
59	76/M	Lung cancer	Prosthesis	90	200	3		24	80
60	73/M	Lung cancer	PF	170	900	4	LR (3 months)	25	83
61	82/M	Gallbladder carcinoma	PF	100	200	6		24	80
62	62/M	Renal cancer	Prosthesis	150	500	3		24	80
63	69/F	Myeloma	Prosthesis	110	300	3		25	83

Patients 5(a, b), 7(a, b), and 21(a, b) each had bilateral humerus fractures

*M* male, *F* female, *IMN* intramedullary nail, *PF* plate fixation, *LR* local recurrence, *FX* fracture, *RNI* radial nerve injury, *ASES* American Shoulder and Elbow Surgeons, *MSTS* Musculoskeletal Tumor Society

Notably, 83.3% (15/18) of the patients with lung carcinoma and 77.8% (14/18) of those with myeloma underwent open reduction and osteosynthesis, while 54.5% (6/11) of the renal cell carcinoma patients underwent segmental resection. Five renal cell carcinoma patients and two thyroid carcinoma metastasis patients did not undergo intercalary prosthetic replacement because of the presence of multiple bone metastases.

The average resection length was 7.8 ± 1.3 cm (range, 6–10 cm) for patients in the prosthesis group. The mean operating duration and blood loss values of the patients treated with intercalary prostheses were smaller than those of the patients who underwent either IMN (116.5 vs. 135 min, *p* = 0.169; 241.2 vs. 539.4 ml, *p* = 0.03) or PF (116.5 vs. 153.3 min, *p* = 0.002; 241.2 vs. 537.9 ml, *p* = 0.012) (Table 3).

**Table 3:** Comparisons among three groups

Variables	IMN (*n* = 16)	PF (*n* = 33)	Prosthesis (*n* = 17)	*P*1 value	*P*2 value	*P3* value
Mean age (yrs)	61.9 ± 12.2	62.1 ± 10.5	63.1 ± 10.5	0.969*	0.766*	0.745*
Follow-up time (mon)	20.8 ± 24.0	18.1 ± 13.1	28.0 ± 25.3	0.666*	0.321*	0.093*
Mean operative time (min)	135 ± 46.4	153.3 ± 36.8	116.5 ± 32.2	0.121*	0.169*	0.002*
Mean blood loss (ml)	539.4 ± 558.5	537.9 ± 370.8	241.2 ± 131.4	0.990*	0.030*	0.012*
Mean ASES score	74.0 ± 6.9	75.6 ± 6.3	76.9 ± 6.8	0.419*	0.206*	0.511*
Mean MSTS score	22.2 ± 2.1	22.7 ± 1.9	23.1 ± 2.1	0.405*	0.214*	0.555*
Complication rate	3/16	7/33	2/17	1.000†	1.000†	1.000†

*P*1: The difference between IMN and PF

*P*2: The difference between IMN and SDR prosthesis

*P*3: The difference between PF and SDR prosthesis

*: Post hoc test

†: Fisher’s exact test

*MSTS* Musculoskeletal Tumor Society

Overall, 12 (12/63, 19%) complications developed after surgery, and 6 of them were treated with another operation. No infections were found in this cohort of patients.

**Fig. 4:**
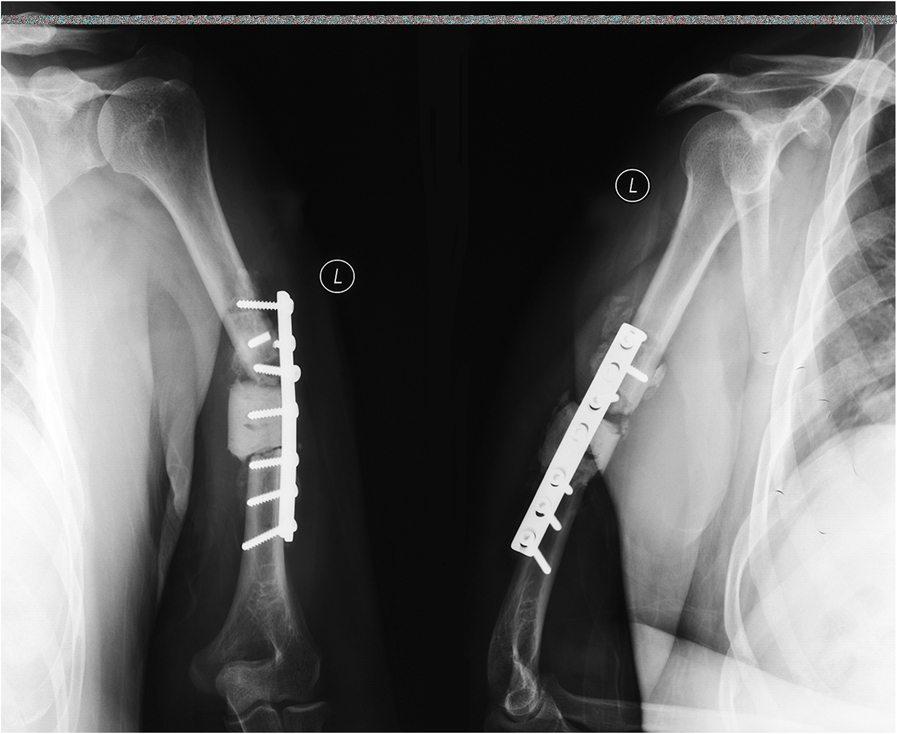
The X-ray film taken 5 months after the initial operation showed plate fixation failure, and revision surgery was performed

In the IMN group, one patient with painful shoulder impingement with functional limitations (patient no. 1) was managed by analgesics and did not undergo additional surgery. Two local recurrences (patient nos. 4 and 7) were observed, resulting in instability and pain. Consequently, patient 4 underwent wide tumor resection and total humeral prosthetic replacement. Patient 7 underwent curettage again and supplementary plate fixation in the distal humerus. In the PF group, temporary iatrogenic radial nerve palsy was found in three cases (patient nos. 14, 38, and 56); fortunately, two patients achieved neurologic recovery within 6 months, while one (patient 14) did not recover, and neurologic deficits remained until the patient’s death. One mechanical failure that occurred 5 months after postoperative radiation (patient no. 12) was attributable to poor initial fixation, and revision surgery with a longer plate was performed (Fig. 4). Two local recurrences (patient nos. 25 and 60) were observed at 6 and 4 months, and both cases were successfully managed by additional resection and prosthetic replacement. Another local relapse (patient no. 54) in the PF group was not treated with another operation due to the deterioration of the patient’s general status.

**Fig. 5:**
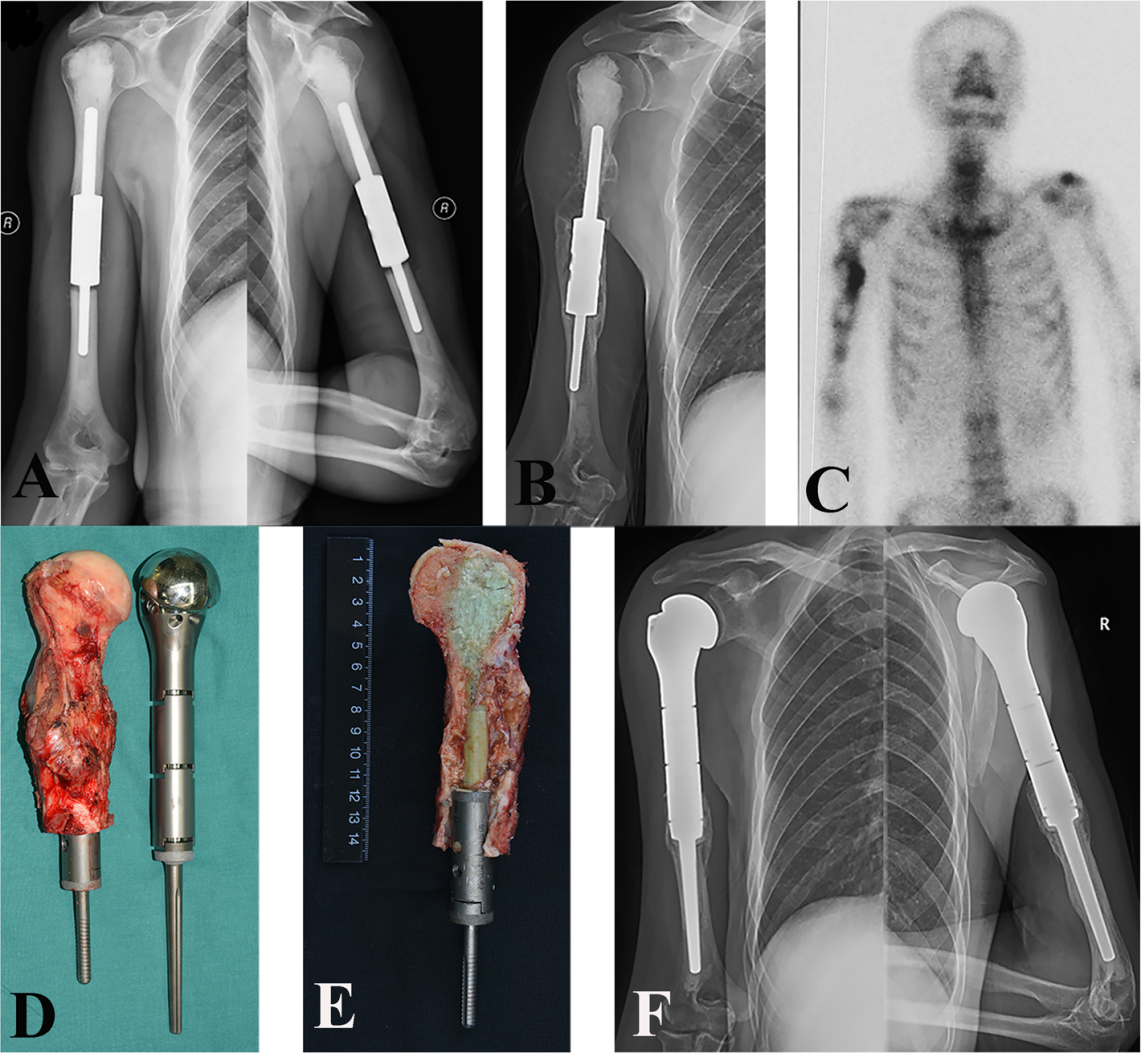
**a** Postoperative radiographs of a 60-year-old man who underwent segmental resection and reconstruction by using a cemented custom-made segmental prosthesis. **b**, **c** The X-ray and bone scintigraphy scan taken 48 months show local relapse proximal to the humerus and loosening of the distal stem. **d**, **e** The intraoperative pictures showed a lesion at the proximal humerus, and then, revision with a modular proximal humeral prosthesis was performed. **f** The postoperative image shows that the position of the prosthesis is satisfactory

**Fig. 6:**
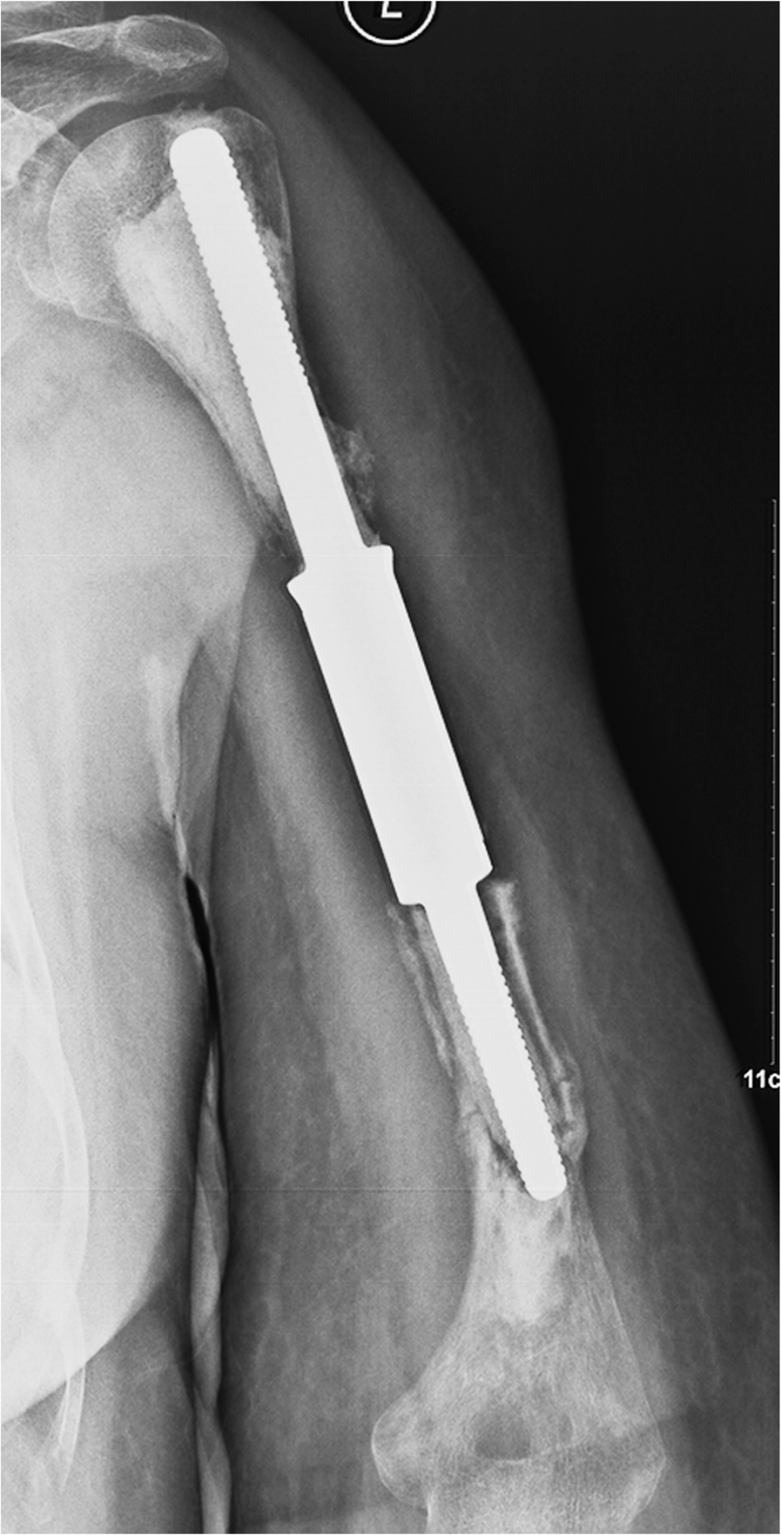
The postoperative radiograph taken 3 years after surgery shows loosening in the distal stem of the prosthesis caused by overuse; however, the function of this limb is still acceptable. The patient did not receive additional surgical management due to the presence of a brain metastasis

In the prosthetic group, one renal cancer patient (patient no. 23) developed local relapse in the proximal humerus 4 years after surgery, and revision with a modular proximal humeral prosthesis was performed (Fig. 5). Another case of aseptic loosening (patient no. 29) that occurred 3 years postoperatively was not treated with additional surgery since a brain metastasis was detected simultaneously (Fig. 6).

At the final follow-up, the mean postoperative upper extremity MSTS scores in the IMN, PF, and prosthetic groups were 22.2 ± 2.1, 22.7 ± 1.9, and 23.1 ± 2.1, respectively, and the mean postoperative ASES scores were 74.0 ± 6.9, 75.5 ± 6.4, and 76.9 ± 6.8, respectively. One-way ANOVA and post hoc analysis did not show any significant differences in functional scores among the three groups.

## Discussion

Currently, prompt pain relief, rapid functional restoration, and good local tumor control are mandatory for most patients with pathologic fractures of the humeral diaphysis, and the use of surgical stabilization will most likely be used more widely in the future. Various surgical options have been described in the literature, with their inherent advantages and disadvantages. The current study presents the results of 66 humerus diaphysis fractures treated with IMN, PF, and intercalary prostheses, aiming to clarify the existing problems and to optimize the treatment strategies. The findings of this study support our hypothesis that prosthetic replacement reduces the operative time, intraoperative blood loss, and postoperative complications more than IMN or PF does and leads to acceptable functional levels.

In this study, the average follow-up time was 20.3 months (range, 3–75 months). A total of 59.7% of the patients lived for more than 1 year, and 46.7% lived for more than 2 years. Prior studies have reported 12-month survival rates of 16% [15], 24% [28], 40% [29], and 88% [30]. The differences in primary tumors, stage of the disease, number of impending fractures, and selection criteria may explain the differences in the survival rate across studies. In this study, we included patients with myeloma or tumors with better prognoses (breast carcinoma, myeloma, renal cell carcinoma, etc.).

Intramedullary nailing, either with or without tumor removal, has been frequently adopted in previous studies [7, 10, 12], although the closed method has the main disadvantage of leaving the tumor mass in situ and making tumor cells spread throughout the medullary cavity. We also performed closed IMN for three patients with bilateral humeral shaft fractures and poor survival estimation. Most patients in this cohort underwent open osteosynthesis with bone cement to enhance the construct and enable patients to withstand the stresses of immediate motions. Intercalary prostheses have been commercially available since 2012, and we started to put use them in clinical practice for select cases. Segmental resection does not require intralesional tumor curettage, and the main blood vessels surrounding the tumor can be ligated before resection. We found that the open IMN and PF groups required longer operation times and had larger volumes of intraoperative hemorrhage due to the intralesional procedure, which is in agreement with the following results reported by Capanna et al. [31]: the “intralesional procedure involving a wide exposure, curettage, filling with cement, and osteosynthesis has no significant merits regarding operating time, blood loss, and recovery time compared with a ‘more aggressive’ wide resection and reconstruction with a prosthesis.”

Twelve postoperative complications were noted in this series. Shoulder movement restrictions after IMN were found in one patient because the extent of antegrade nailing was inadequate. One case of plate failure occurred and was resolved by replating; therefore, we believe that poor initial fixation might have been avoided if a longer plate had been used. This is an example of the many pitfalls of incorrectly using locking plate technology. Compared with prosthetic replacement, the osteosynthetic method generally provides partly local tumor control. All local tumor recurrences in the patients who underwent IMN and PF fixation developed within 1 year in this study. The incidence of iatrogenic radial nerve injury after plate fixation for humerus fractures varies widely and ranges from 6 to 16% [28, 29, 32, 33]. In this study, 3 (9.1%) nerve injuries developed on account of extensive soft-tissue stripping in patients in the PF group with lesions located at the middle-lower 1/3 of the humerus, and this condition tended to lead to radial nerve injury. We are not sure whether iatrogenic radial nerve palsy could have been avoided had we chosen to perform retrograde IMN. In the procedure of intercalary prosthetic replacement, we were inclined to shorten the humerus by appropriately 1 to 2 cm to decrease the risk of overstretch injury to the radial nerve and to obtain better soft tissue coverage for the prosthesis. None of the patients in this group experienced nerve palsy.

It is unclear whether IMN leads to better outcomes than does PF. In a study conducted by Wedin et al., intramedullary nails failed in 7% of patients, and plate fixation failed in 22% [34]. However, Dijkstra et al. found that IMN and plate fixation yielded similar results [15]. In the present study, the incidence of postoperative complications in the IMN group was 18.8% (3/16) and 21.2% (7/33) in the PF group, which were in the range of reported incidence rates in previous studies [8, 9, 13–15, 19, 20, 28, 34–36] (Table 4).

**Table 4:** Comparison of current study results with those of other studies involving intercalary endoprostheses

Author, year	Case number	Average age (years)	Treatment	Complication	Mean follow-up time (months)	Mean MSTS (%)
Dijkstra et al. [15], 1996	38	65	IMN (18)	Fracture: 1 Instability: 2	/	/
			PF (20)	Wound problem: 2 Nerve injury: 1 Local recurrence: 1	/	/
Ofluoglu et al. [9], 2009	24	63	IMN	Loosening: 2	/	/
Sarahrudi et al. [28], 2009	41	66.3	IMN (19)	Instability: 2 Tumor progression: 1	/	/
		70.2	PF (22)	RNI: 4 Fracture: 1 Loosening: 1	/	/
Ruggieri et al. [36], 2011	8	/	Prosthesis	Loosening: 1	25	90
McGrath et al. [8], 2011	13	35	Endoprosthesis	Local recurrence: 2 Loosening: 4 Fracture: 2 Nerve injury: 2	57	77
Laitinen et al. [35], 2011	40	65	IMN	7 (18%)	/	70/68
Wedin et al. [34], 2012	128	/	IMN (117)	Fracture: 1 Nonunion: 5 Infection: 2	/	/
		/	PF (11)	Fracture: 2	/	/
Benevenia et al. [13], 2016	18	69	Prosthesis	None	10	83
Zhao et al. [19], 2018	9	63	Prosthesis	RNI: 1 Aseptic loosening: 1	9	85
Casadei et al. [14], 2018	12	67	IMN	Infection: 1 Local recurrence: 1 Loosening: 2	/	63
Zheng et al. [20], 2019	13	/	Prosthesis	RNI: 2 Aseptic loosening: 2	11	65
Current study	66	62	IMN (16)	Local recurrence: 2 Rotator cuff injury: 1	21	74
		61	PF (33)	RNI: 3 Local recurrence: 3 Screw break: 1	18	76
		63	Prosthesis (17)	Local recurrence: 1 Aseptic loosening: 1	28	77

*IMN* intramedullary nailing, *PF* plate fixation, *RNI* radial nerve injury, *MSTS* Musculoskeletal Tumor Society score

Admittedly, the use of prostheses cannot prevent the occurrence of complications in the long term. Aseptic loosening of the prosthetic stem occurred in a prolonged breast cancer survivor, possibly due to overuse of the involved upper limb. Another renal cell carcinoma patient experienced local tumor recurrence 4 years after the operation.

**Fig. 7:**
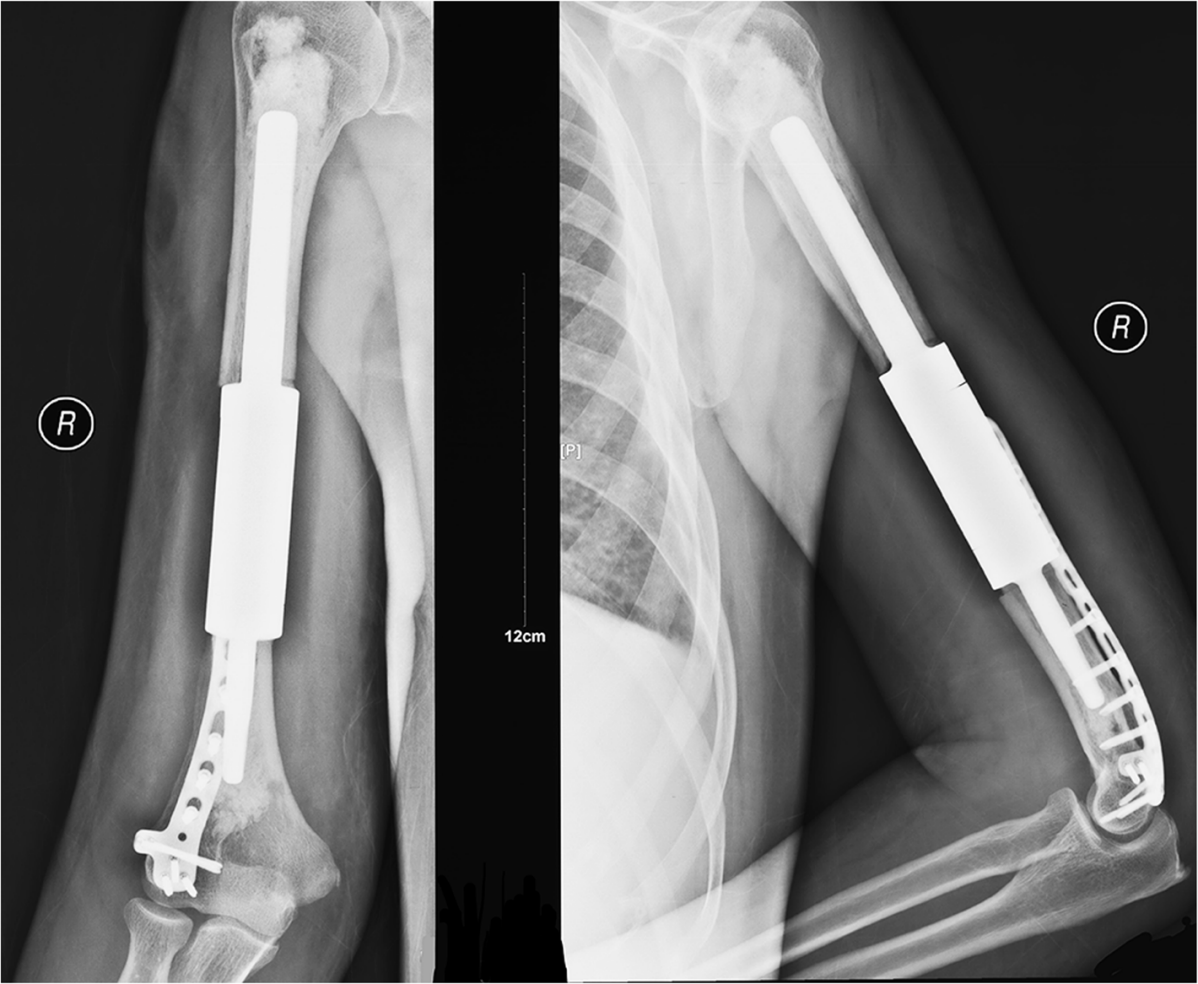
The postoperative X-ray film shows that an additional extracortical plate was applied when the fixation length was less than 5 cm

Abudu et al. concluded that the possibility of early loosening is higher when the intramedullary fixation length of the intercalary prosthesis is shorter than 5 cm [11]. To avoid this problem, we applied an additional extracortical plate in one patient with a distal stem of 4 cm to reduce mechanical torsional stresses at the bone-stem interface (Fig. 7).

Ruggieri et al. [36] presented 8 cases with metastatic humeral lesions treated with intercalary prostheses. The mean follow-up time was 24.9 months (range, 7–50), and only one case of mechanical loosening was found at 30 months. Benevenia et al. [13] applied 17 intercalary endoprostheses for metastatic humeral shaft lesions, and there were no complications during the 9.5-month follow-up period. The authors recommended that the use of intercalary endoprostheses is a reasonable alternative to consider.

We observed that the average MSTS and ASES scores, indicating function, of the intercalary prosthetic replacement patients were 23.1 and 76.9, respectively, and these values were comparable to those of the IMN and PF groups. Overall, intercalary prosthetic replacement seemed to yield acceptable levels of function and better local disease control, most notably with respect to the reduced postoperative time and blood loss.

**Fig. 8:**
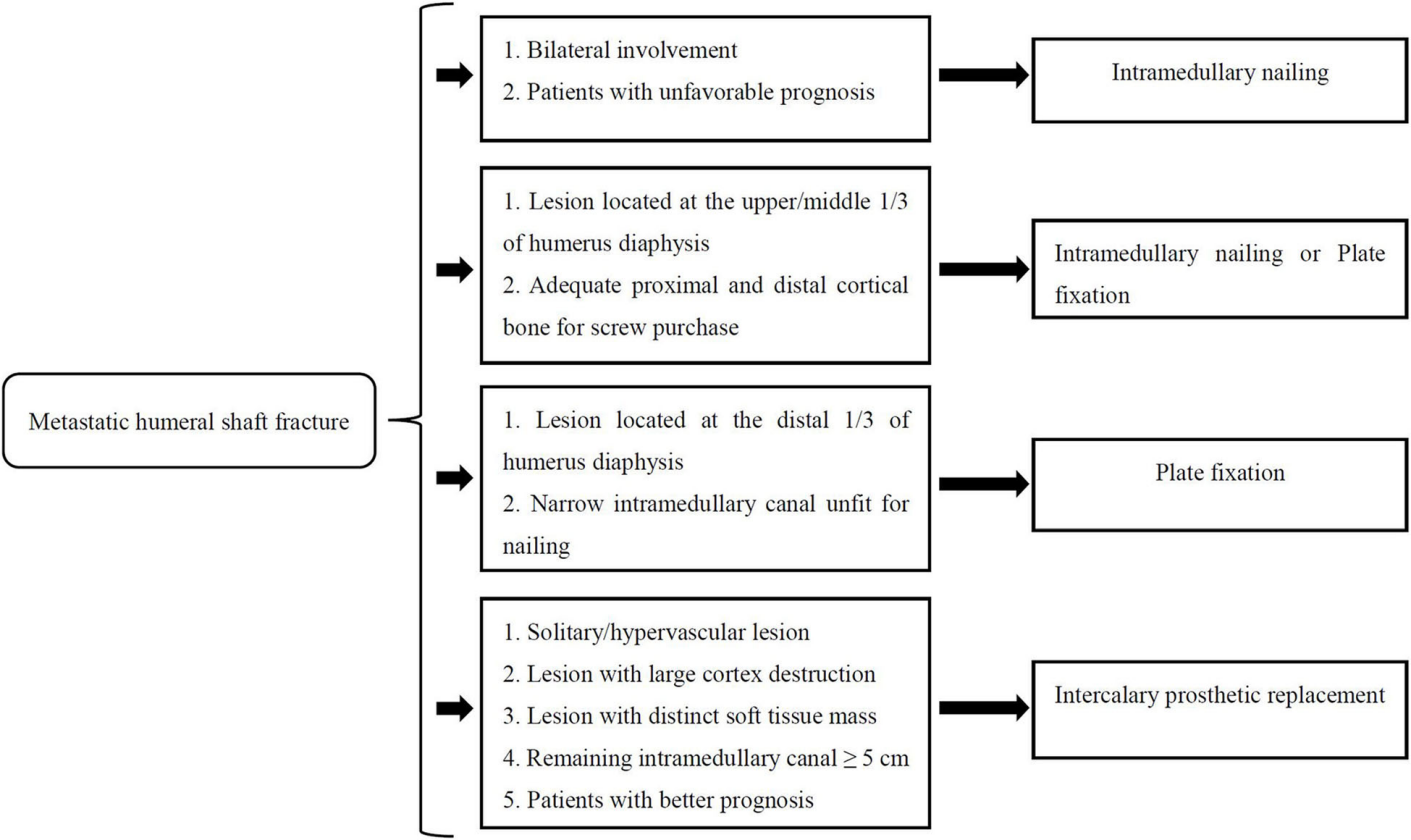
Map of suggested elements for the surgical treatment of metastatic humeral shaft fractures

In recent years, an increasing number of studies have reported different reconstructive strategies for humeral diaphysis metastases with varied outcomes, depending on the experience of each team [1, 7, 10, 11, 13, 14, 18, 19]. The optimal implant choice requires the consideration of both patient- and surgeon-controlled factors. Surgical treatment is usually tailored on a patient-to-patient basis taking into account certain standard recommendations. Most patients are still treated with IMN or plates according to published studies [15, 28, 29], and the present study corroborates this trend: most patients (74.2%) underwent osteosynthesis, but intercalary prostheses were also used. Admittedly, the indications for these three surgical procedures were not mutually exclusive. Fewer complications and better functional outcomes will be obtained when surgical indications are followed correctly, leading to marked improvement in patient quality of life. In order to individualize the treatment (IMN, PF, or prosthesis) to different humeral metastasis, we formulate a pathway that may help us to choose the surgical technique (Fig. 8).

Some limitations of this study require discussion. First, this was an observational case series study, and due to its retrospective and nonrandomized nature, selective bias and confounding bias may have been present in the study. Second, the type of resection and reconstruction was affected by the year of treatment and by patient and tumor factors. The intercalary prosthesis became available after 2012, and to some extent, patients with better prognoses were more likely to undergo segmental prostheses. Thus, comparative conclusions may not be appropriate. We believe that additional randomized prospective studies comparing these techniques may get more convincing results. Nonetheless, even with these limitations, our results may be useful.

## Conclusion

Three stabilization methods are effective in the treatment of humeral diaphysis metastatic lesions. Compared with IMN and PF, segmental endoprosthesis replacement may provide a construct associated with a shorter operative duration, less blood loss, lower complication rates, and comparable functional results. It may serve as an optimal stabilization method in exceptional situations with extensive cortical destruction or widespread soft-tissue extension or for those patients with better prognosis. Intercalary prostheses will most likely be used more widely in the future.

## Supplementary Information

**Additional file 1: Table I.** The additional details of patients.

## Data Availability

The datasets used and/or analyzed during the current study are available from the corresponding author on reasonable request.

## Abbreviations

IMN: Intramedullary nailing
PF: Plate fixation
KPS: Karnofsky performance score
VAS: Visual analogue scale
ASES: American Shoulder and Elbow Surgeons
MSTS: Musculoskeletal Tumor Society
SD: Standard deviation
