# Supplementary material for: Intercalary prosthetic replacement is a reliable solution for metastatic humeral shaft fractures: retrospective, observational study of a single center series
Source: World J Surg Oncol. 2021 May 5;19:140. doi: 10.1186/s12957-021-02250-1 (PMC8101207; doi:10.1186/s12957-021-02250-1)
Supplement: Supplementary file 1 — Additional file 1: Table I. The additional details of patients. [file 12957_2021_2250_MOESM1_ESM.docx]

**Table I** The details of patients

| **No.** | **Interval**  **(month)** | **Location** | **Fracture** | **Soft-tissue mass** | **Visceral metastasis** | **KPS** | **VAS** | **Mirel’s score** | **TNM stage** |
| --- | --- | --- | --- | --- | --- | --- | --- | --- | --- |
| 1 | 12 | Middle 1/3 | Complete | No | Brain | 50 | 8 | / | T2aN1M1 |
| 2 | 0 | Middle 1/3 | Complete | No |  | 80 | 6 | / | / |
| 3 | 0 | Middle 1/3 | Complete | Yes |  | 70 | 7 | / | / |
| 4 | 0 | Middle 1/3 | Impending | No |  | 70 | 5 | 8 | T1aN0M1 |
| 5a | 11 | Middle 1/3 | Impending | No |  | 40 | 8 | 9 | / |
| 5b | 11 | Middle 1/3 | Complete | No |  |  | 8 | / |  |
| 6 | 0 | Middle 1/3 | Impending | Yes |  | 80 | 5 | 8 | T1bN0M1 |
| 7a | 6 | Upper 1/3 | Complete | No | Brain | 50 | 7 | / | / |
| 7b | 6 | Upper 1/3 | Complete | No |  |  | 5 | / |  |
| 8 | 8 | Middle 1/3 | Complete | No | Lung; Liver | 70 | 9 | / | TxN0M1 |
| 9 | 0 | Middle 1/3 | Complete | No |  | 70 | 9 | / | TxNxM1 |
| 10 | 24 | Middle 1/3 | Impending | Yes | Liver | 80 | 5 | 8 | TxNxM1 |
| 11 | 12 | Middle 1/3 | Impending | Yes |  | 70 | 6 | 9 | T3N0M1 |
| 12 | 0 | Middle 1/3 | Complete | No | Lung | 60 | 8 | / | T1cN1M1 |
| 13 | 0 | Upper 1/3 | Complete | Yes |  | 80 | 8 | / | TxNxM1 |
| 14 | 0 | Lower 1/3 | Complete | No |  | 50 | 8 | / | T1aN1M1 |
| 15 | 84 | Middle 1/3 | Impending | No | Brain | 50 | 4 | 8 | T2N1M1 |
| 16 | 15 | Lower 1/3 | Complete | Yes |  | 90 | 4 | / | TxNxM1 |
| 17 | 0 | Upper 1/3 | Impending | No | Lung | 50 | 4 | 8 | T2N1M1 |
| 18 | 24 | Middle 1/3 | Impending | Yes | Lung | 70 | 3 | 9 | TxN1M1 |
| 19 | 36 | Middle 1/3 | Complete | No | Lung | 30 | 7 | / | / |
| 20 | 0 | Middle 1/3 | Complete | Yes |  | 80 | 8 | / | TxNxM1 |
| 21a | 24 | Upper 1/3 | Impending | No |  | 70 | 5 | 8 | T3N1M1 |
| 21b | 24 | Upper 1/3 | Complete | No |  |  | 8 | / |  |
| 22 | 0 | Middle 1/3 | Impending | Yes |  | 90 | 5 | 8 | TxN0M1 |
| 23 | 0 | Middle 1/3 | Complete | Yes |  | 30 | 7 | / | T1bN1M1 |
| 24 | 72 | Middle 1/3 | Complete | Yes |  | 80 | 6 | / | TxNxM1 |
| 25 | 0 | Middle 1/3 | Complete | No |  | 70 | 8 | / | T2N0M1 |
| 26 | 0 | Middle 1/3 | Complete | Yes |  | 90 | 5 | / | TxNxM1 |
| 27 | 0 | Middle 1/3 | Impending | No |  | 70 | 8 | 9 | / |
| 28 | 9 | Middle 1/3 | Impending | Yes |  | 90 | 4 | 8 | T3N1M1 |
| 29 | 84 | Middle 1/3 | Impending | No |  | 90 | 7 | 8 | TxN0M1 |
| 30 | 48 | Middle 1/3 | Impending | No |  | 70 | 8 | 9 | / |
| 31 | 0 | Middle 1/3 | Complete | No |  | 70 | 6 | / | T3N1M1 |
| 32 | 0 | Middle 1/3 | Impending | Yes |  | 60 | 7 | 8 | / |
| 33 | 0 | Middle 1/3 | Complete | No |  | 80 | 8 | / | / |
| 34 | 14 | Middle 1/3 | Impending | Yes |  | 80 | 7 | 9 | TxNxM1 |
| 35 | 84 | Middle 1/3 | Impending | Yes |  | 70 | 5 | 8 | T1aN0M1 |
| 36 | 0 | Lower 1/3 | Complete | No |  | 90 | 4 | / | T1N0M1 |
| 37 | 48 | Middle 1/3 | Complete | No |  | 80 | 7 | / | TxNxM1 |
| 38 | 120 | Upper 1/3 | Complete | Yes |  | 70 | 8 | / | T2N0M1 |
| 39 | 0 | Lower 1/3 | Complete | No |  | 40 | 9 | / | / |
| 40 | 0 | Middle 1/3 | Impending | No | Lung | 50 | 8 | 9 | T1cN0M1 |
| 41 | 0 | Upper 1/3 | Complete | Yes |  | 70 | 4 | / | / |
| 42 | 13 | Middle 1/3 | Complete | No |  | 60 | 7 | / | T2N1M1 |
| 43 | 0 | Middle 1/3 | Impending | Yes |  | 90 | 8 | 9 | / |
| 44 | 0 | Upper 1/3 | Complete | No |  | 70 | 7 | / | T3N1M1 |
| 45 | 36 | Middle 1/3 | Impending | No | Lung | 90 | 5 | 8 | TxN0M1 |
| 46 | 3 | Upper 1/3 | Complete | No |  | 50 | 4 | / | / |
| 47 | 0 | Middle 1/3 | Complete | Yes | Lung | 40 | 7 | / | T4N1M1 |
| 48 | 24 | Middle 1/3 | Complete | No |  | 60 | 8 | / | / |
| 49 | 0 | Middle 1/3 | Impending | No |  | 90 | 8 | 9 | T1aN0M1 |
| 50 | 0 | Upper 1/3 | Impending | No |  | 80 | 4 | 8 | / |
| 51 | 0 | Middle 1/3 | Impending | No |  | 80 | 5 | 8 | / |
| 52 | 0 | Middle 1/3 | Complete | Yes | Lung | 60 | 8 | / | T2N0M1 |
| 53 | 12 | Middle 1/3 | Complete | No |  | 80 | 7 | / | / |
| 54 | 0 | Lower 1/3 | Complete | Yes | Adrenal gland | 60 | 8 | / | T3N1M1 |
| 55 | 4 | Middle 1/3 | Impending | Yes |  | 90 | 7 | 9 | T3N0M1 |
| 56 | 23 | Middle 1/3 | Complete | Yes | Liver | 50 | 6 | / | TxN1M1 |
| 57 | 0 | Middle 1/3 | Impending | No |  | 30 | 4 | 8 | / |
| 58 | 0 | Lower 1/3 | Impending | Yes |  | 90 | 5 | 8 | T1cN0M1 |
| 59 | 0 | Middle 1/3 | Complete | Yes | Liver | 60 | 3 | / | T3N1M1 |
| 60 | 0 | Middle 1/3 | Complete | No |  | 70 | 3 | / | T3N1M1 |
| 61 | 0 | Middle 1/3 | Complete | Yes | Liver | 80 | 4 | / | T2N0M1 |
| 62 | 0 | Middle 1/3 | Complete | Yes |  | 80 | 5 | / | T3N0M1 |
| 63 | 0 | Middle 1/3 | Complete | Yes |  | 80 | 8 | / | / |

Patients 5(a, b), 7(a, b), and 21(a, b) each had bilateral humerus fractures;

M = Male; F = Female; Interval: The time span between the diagnosis of primary tumor and the development of humeral shaft metastasis; IMN = Intramedullary nail; KPS: Karnofsky performance score; PF: Plate fixation; VAS: Visual Analogue Scale/Score;
